# Elevated CO_2_ Affects Predator-Prey Interactions through Altered Performance

**DOI:** 10.1371/journal.pone.0058520

**Published:** 2013-03-06

**Authors:** Bridie J. M. Allan, Paolo Domenici, Mark I. McCormick, Sue-Ann Watson, Philip L. Munday

**Affiliations:** 1 ARC Centre of Excellence for Coral Reef Studies, and School of Marine and Tropical Biology, James Cook University, Townsville, Queensland, Australia; 2 CNR-IAMC, Istituto per l’Ambiente Marino Costiero, Località Sa Mardini, Torregrande, Oristano, Italy; The Australian National University, Australia

## Abstract

Recent research has shown that exposure to elevated carbon dioxide (CO_2_) affects how fishes perceive their environment, affecting behavioral and cognitive processes leading to increased prey mortality. However, it is unclear if increased mortality results from changes in the dynamics of predator-prey interactions or due to prey increasing activity levels. Here we demonstrate that ocean *p*CO_2_ projected to occur by 2100 significantly effects the interactions of a predator-prey pair of common reef fish: the planktivorous damselfish *Pomacentrus amboinensis* and the piscivorous dottyback *Pseudochromis fuscus*. Prey exposed to elevated CO_2_ (880 µatm) or a present-day control (440 µatm) interacted with similarly exposed predators in a cross-factored design. Predators had the lowest capture success when exposed to elevated CO_2_ and interacting with prey exposed to present-day CO_2_. Prey exposed to elevated CO_2_ had reduced escape distances and longer reaction distances compared to prey exposed to present-day CO_2_ conditions, but this was dependent on whether the prey was paired with a CO_2_ exposed predator or not. This suggests that the dynamics of predator-prey interactions under future CO_2_ environments will depend on the extent to which the interacting species are affected and can adapt to the adverse effects of elevated CO_2_.

## Introduction

Predation is one of the key processes structuring communities in ecological and evolutionary time [1]. Prey with well-honed antipredator responses will have high survival, while predators with low catch rates suffer slower growth and reduced reproductive output [2], [3]. Any factor that influences a part of the predator-prey interaction sequence will affect the outcome for both parties. Recent research has shown that carbon dioxide (CO_2_) levels projected to occur in the ocean by the end of this century (based on Representative Concentration Pathways RCPs) [4] can affect the behavior of marine organisms and significantly increase predation rates in natural habitat [5]–[10]. These findings emphasize the potential impact that elevated CO_2_ in the ocean could have on marine population dynamics and ecosystem processes [11]–[13]. To date however, the dynamic mechanisms of predator-prey interactions that underlie increased predation rates in high CO_2_ environments are unknown.

The concentration of CO_2_ in the atmosphere has increased approximately 40% since the industrial revolution, to present-day levels >390 ppm. The atmosphere and surface-ocean are in approximate gas equilibrium; therefore the concentration of CO_2_ in the ocean also increases as atmospheric CO_2_ increases [14]. If the current trajectory of emissions is maintained, atmospheric CO_2_ concentrations are predicted to exceed 900 ppm by the year 2100 [4]. It has recently been demonstrated that these levels of dissolved CO_2_ can dramatically alter the response of fish predators to prey sensory cues [15] and of fish prey to the presence of predators [6]. Fish exposed to elevated CO_2_ exhibit impaired olfactory [6], [15] and auditory responses [16], altered activity levels [7], [9] and reduced behavioral lateralization (the propensity to turn left or right) [17]. The underlying mechanism for these behavioural changes appears to be impaired neurotransmitter function in fish that are permanently exposed to elevated CO_2_ [18]. If elevated CO_2_ alters the processing of sensory information, then it may directly influence the behavioral basis that governs the outcome of a predator-prey interaction, such as the timing of a predator strike or the distance to react to the predator by the prey. During a predator-prey interaction, fish employ a rapid acceleration called a fast-start that is used by predators to capture prey, or by prey to avoid a predatory strike [19], [20], [21]. Consequently, any effect of elevated CO_2_ on the timing or performance of fast-starts by either the predator or prey could lead to changes in prey mortality.

This study examined the potential cause of increased mortality of prey fish that has been observed in previous studies [7], [9] by asking the specific question: Does exposure to elevated CO_2_ change the outcome of predator-prey encounters by altering the kinematics of the predator-prey interaction? To test this hypothesis, locomotion performance, prey reaction distance and capture success were examined in staged encounters between newly metamorphosed individuals of a prey fish, *Pomacentrus amboinensis*, and a common predator, *Pseudochromis fuscus*. Predators and prey were exposed to CO_2_ levels (880 µatm) relevant to the end of the century based on the most recent representative greenhouse gas concentration pathways [4]. Previous experiments have demonstrated that the mortality rates of high-CO_2_ exposed prey when placed in the field with non-CO_2_ exposed predators are 2–3 times higher than when prey are exposed to similarly treated predators in the laboratory [7], [9]. This suggests that the behavior of the CO_2_ exposed prey depends on whether or not the predator has also been exposed to elevated CO_2_. Therefore, the responses of fish exposed to high CO_2_ were compared to fish exposed to present day CO_2_ levels (440 µatm) in a fully crossed design. This enabled us to tease apart the independent effects on the predator and prey as well as the interacting effects when both were exposed to elevated CO_2_.

## Materials and Methods

### Ethics Statement

Research was carried out under approval of the James Cook University animal ethics committee (permit: A1067) and according to the University’s animal ethics guidelines. Fish collections around Lizard Island, Great Barrier Reef were carried with permission of the Great Barrier Reef Parks Authority (permit: G10/33239.1) and Queensland Government Department of Primary Industry and Fisheries (permit: 103256). Suffering was minimal as prey were consumed immediately following a successful strike.

### Study Site and Species

Fishes were collected during December 2010 at Lizard Island (14° 40′ S, 145° 28′ E), northern Great Barrier Reef (GBR) and maintained in a flow-through seawater system at the Lizard Island Research Station (LIRS). Newly metamorphosed individuals of the common damselfish, *Pomacentrus amboinensis* (Pomacentridae) were used as the prey species. The dottyback, *Pseudochromis fuscus* (Pseudochromidae) was used as the predator. *P. fuscus* is an abundant, small, widely distributed mesopredator found throughout the Indo-Pacific. It is a gape limited, highly territorial and active predator and makes up 9.5% of the piscivorous reef fish assemblage at Lizard Island [22]. It is considered an important predator of newly settled coral reef fishes [23], [24]. *P. fuscus* readily adjusts to aquarium conditions and has been observed exhibiting normal feeding and aggressive behaviors within 48 hours of collection [25]. Because of this, it has been used extensively as a model predator in predator-prey manipulation studies.

Newly metamorphosed *P. amboinensis* (range 10.3–15.1 mm, 12.6 mean standard length (SL), standard deviation (SD) 1.5) [26] were collected using light traps [27] moored ∼100 m off the fringing reef of Lizard Island. On the morning of capture they were transferred to 30 L aquaria supplied with a continuous flow of either control (present-day CO_2_) or elevated-CO_2_ seawater (see below) for 4 days. This period of time has been found to be sufficient to elicit the full behavioural effects of high CO_2_ and larval fish do not become acclimated with longer exposure [28]. Fish were fed 4 times daily *ad libitum* with newly hatched *Artemia* sp. but were starved for the 12 hours prior experimental trials to standardize for satiation.

Adult *P. fuscus* (range 64–83 mm, 72.3 mm mean SL, SD 0.6) were collected with a dilute solution of clove oil [29] from of the shallow fringing reef around Lizard Island. Immediately after collection, fish were transported back to LIRS where they were housed separately in mesh breeding baskets within 30 L aquaria to avoid aggressive interactions. Fish were maintained in tanks for 4 days and were fed 2 juvenile reef fish for the first 2 days and then not fed for the last 2 days to standardize for satiation. Food deprivation in the wild is not unusual and previous work has demonstrated a high prevalence of gut emptiness for piscivorous fish [30].

### CO_2_ Treatment

The *p*CO_2_ of treatment seawater was manipulated by CO_2_ dosing to a set pH_NBS_. Seawater was pumped from the ocean into 2×60 L sumps where it was diffused with ambient air (control) or CO_2_ to achieve the desired pH (CO_2_ treatment). A pH of 7.89 was selected to achieve the approximate *p*CO_2_ required, based on preliminary observations of total alkalinity, salinity and temperature of seawater at Lizard Island. A pH-controller (Tunze Aquarientechnik, Germany) was attached to the CO_2_ treatment sump to maintain pH at the desired level. A solenoid injected a slow stream of CO_2_ into a powerhead at the bottom of the sump whenever the pH of the seawater rose above the set point. Equilibrated seawater from each sump was then supplied at a rate of ∼500 ml.min^−1^ to eight replicate 35 L aquariums, four housing small groups of *P. amboinensis* and four housing *P. fuscus*. Temperature and pH_NBS_ of each aquarium was measured each morning and afternoon using an HQ40d pH meter (Hach, Colorado, USA) calibrated with fresh buffers. Total alkalinity (TA) of seawater was estimated by Gran titration from water samples taken twice weekly from control and treatment tanks. Alkalinity standardizations achieved accuracy within 1% of certified reference material from Dr. A. Dickson (Scripps Institution of Oceanography). Average seawater *p*CO_2_ was calculated using measured values of pH, TA, temperature and salinity in the program CO2SYS [31] and using the constants of Mehrbach *et al*. [32] refit by Dickson and Millero [33]. Seawater parameters are shown in Table 1.

**Table 1:** Mean (±SD) seawater parameters in the experimental system.

pH_NBS_	temp (°C)	salinity (ppt)	TA (µmol.kg^−1^SW)	pCO_2_(µatm)
8.15 (0.04)	27.66 (0.98)	35	2269.66 (15.01)	440.53 (44.46)
7.89 (0.06)	27.74 (0.99)	35	2261.23 (14.92)	879.95 (140.64)

Temperature, pH salinity, and total alkalinity (TA) were measured directly. *p*CO_2_ was estimated from these parameters using CO2SYS.

### Laboratory Assays

Trials were conducted over a period of 10 days in a temperature-controlled room at LIRS, ensuring the water temperature remained between 26 and 28°C. One predatory *P. fuscus* was placed into the experimental arena (38 cm×58 cm×10 cm water height) and one *P. amboinensis* was then released into a length of PVC tube (11 cm diameter, 15 cm high) placed upright in the middle of the experimental arena. Both fish were allowed to acclimate for 30 min. The PVC tube was then carefully raised and removed from the tank using a wire connected to the top of the tube. This allowed the predator and the prey to start the interaction.

A soundproof polystyrene lid was placed on the experimental arena to minimize disturbance and to eliminate observer effect. A high-speed video camera (Casio ex-fh20; 420 fps) recorded fish behavior through a hole in the lid and trials were filmed until the prey was consumed or 10 min had elapsed. The water in the experimental arena was changed following each trial to maintain oxygen saturation. SL (defined as the length of a fish measured from the tip of the snout to the posterior end of the last vertebra) of the predator and prey and the water temperature were recorded for each trial.

In order to partition the relative effects of CO_2_ on predators, prey and the full interaction, four combinations of CO_2_-treated and control predators and prey were undertaken in a crossed design: control predator vs control prey (n = 21); treated predator vs treated prey (n = 21); control predator vs treated prey (n = 16); treated predator vs control prey (n = 16). Predator and prey fish were used only once in each trial. This ensured both were naïve to the experimental procedure. All combinations of treatments were undertaken daily to control for any potential daily variation. Video-analysis could not be performed on all interaction trials, due to fish leaving the screen at the time of the response and/or technical problems with the video. As a result, the sample size varied slightly among the performance traits measured. Trials were only used when the predator was at the opposite end of the tank to the prey at the start of the interaction. This was done to standardize for predator position. Maximum predator attack speed and maximum prey escape speed (UMAX_pred_ and UMAX_prey_) were measured based on the center of mass (COM) of the fish when stretched straight based on Webb [34]. COM was assumed to be at 35% of the body length from the tip of the snout as it is the case for generalist fish [31]. Speed was smoothed using a 5-point differentiation-based moving polynomial regression [35]. Stage 1 and 2 where defined based upon directional changes of the anterior part of the body of the fish, based on Domenici and Blake [19].

Prey escape variables were measured only when prey performed a C-start. Predator attacks were measured only when a predator showed a fast-directed burst towards the prey (>3 body lengths s^−1^). All variables with the exception of number of prey caught were measured using only the first attack that occurred within a trial. This was done to control for any anaerobic stress either the predator or prey may have experienced due to prolonged attacks.

### Kinematic Analysis

Video recordings were analyzed using WinAnalyze motion-analysis software (v. 1.9 2D; Mikromak Service Brinkmann, Berlin, Germany). In each frame the snout and the COM (center of mass) were located on each fish. These points were chosen to standardise each frame.

The following performance variables were measured:

### Predator

1. Capture success: percentage of trials in which the predator ingested the prey within the 10 min filming period, out of the total number of trials for each treatment.
2. Attack rate: number of attacks per unit time, measured for each interaction.
3. Predation rate: capture success divided by the number of attacks per unit time.
4. Predator attack distance (D_pred_; m): the straight-line distance between the predator centre of mass (COM) at the time the attack commenced and the end of the attack. The end of the attack is defined as when the predator came to a halt.
5. Maximum predator attack speed (UMAX_pred_; m s^−1^): the top speed achieved at any point in time during the attack, based on the predator COM (see electronic supplemental material).

### Prey

1. Prey reaction distance (R_D_; m): the distance between the prey COM and the tip of the predator’s snout at the onset of the escape response to a predator attack.
2. Apparent Looming Threshold (ALT) is defined as the apparent looming threshold for prey avoidance responses to a predatory strike and is a measure of the magnitude of the preys response to the perceived threat of predation. The higher the perceived threat, the higher the angle, (ALT; radians s^−1^): measured at the onset of the escape response and measured as the rate of change of the angle (α) subtended by the predator’s frontal profile as seen by the prey. Previous work has shown that fish tend to react to an approaching stimulus (a predator) when a given threshold of dα/dt (i.e. ALT) is reached. ALT is calculated as (4US)/(4D^2^+S^2^), based on Dill [36] and Webb [37]. where U = predator speed, calculated as the speed of the predator in the frame prior to the prey’s response; S = (Max. Depth+Max. Width)/2, where Max. Depth (DMAX) was estimated to be positioned at one-quarter of the body length of the predator (L_pred_) (pers. obs.) and maximum width (WMAX) at 0.25L_pred_; D = R_D_ +0.25L_pred_.
3. Prey escape distance (D_prey_; m): the straight-line distance between the prey COM at the onset of the escape response and at the end of the escape response (i.e. when the prey comes to a halt).
4. Maximum prey escape speed (UMAX_prey_; m s^−1^): the top speed achieved at any point in time during the escape response, measured using the prey COM.
5. Mean prey escape distance during stage 1 & 2 (Ds1s2; m): the distance between the COM of the fish at frame 0 and 24 ms later. This fixed duration was based on the average duration (22.8±3.2 ms), of the first two tail flips of the tail (the first two axial bends, i.e. stages 1 and 2 defined based on [19], which is the period considered crucial for avoiding ambush predator attacks [19], [34].

### Statistical Analyses

To test the null hypothesis that feeding success is independent of predator and prey CO_2_ exposure, predator success was compared by 2×4 contingency table analysis. The effects of CO_2_ elevation on predator-prey interactions were examined using a 2-factor MANOVA with the fixed factors: Prey treatment (Control and CO_2_ elevated) and Predator treatment (Control and CO_2_ elevated). Univariate 2-factor ANOVAs with Tukey’s HSD post-hoc tests were performed to determine the nature of any differences found by MANOVA. Predation rate data was arcsine transformed to meet the assumption of homogeneity of variance. Residual analysis indicated that data met the assumptions of normality and homogeneity of variance.

## Results

**Figure 1:**
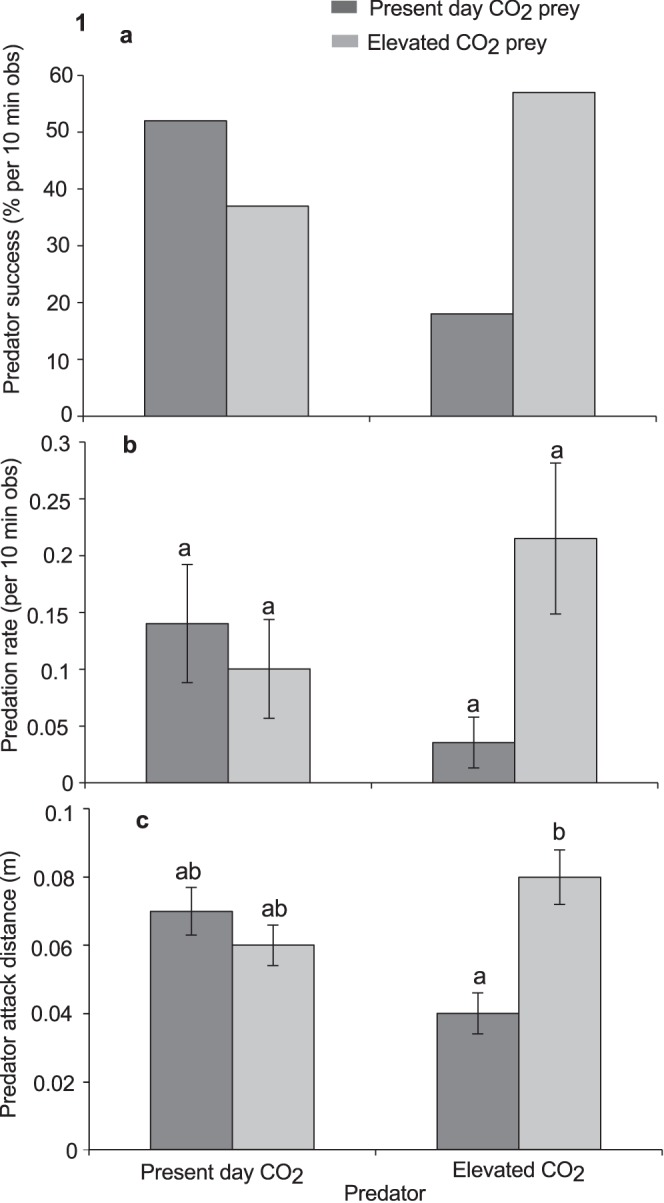
Effects of elevated CO_2_ on predator attack performance. Comparison of the effects of elevated CO_2_ (440, 880 µatm) on interactions between a predator (*Pseudochromis fuscus*) and prey (*Pomacentrus amboinensis*) on 3 performance variables of the predator: (a) predator success (b) predation rate and (c) predator attack distance. (a) N = 21, 16, 16, 21, (b) N = 21, 16, 16, 21 and (c) N = 17, 15, 12, 16 (left to right). Errors are standard errors. Letters above bars represent Tukey’s HSD groupings of means.

Capture success was significantly associated with the CO_2_ exposure treatment experienced by the predator and prey (χ^2^ = 8.95, df = 3, p = 0.03; Fig. 1a). When both the predator and prey had been exposed to elevated CO_2_ the capture rate (52%) was similar to that of the control group where both predators and prey were exposed to present-day CO_2_ (51%). In contrast, the capture rate of these two treatment groups was markedly higher than the CO_2_ predator-Control prey group. When predators exposed to elevated CO_2_ were given prey exposed to present-day CO_2_, capture success of the predator was 33% less than the treatment groups where both predators and prey had been exposed to the same levels of CO_2_ (either elevated or present-day CO_2_). Capture success was also 14% less for predators exposed to present-day CO_2_ with elevated CO_2_ exposed prey compared with treatment groups where both predators and prey had been exposed to the same levels of CO_2_.

The 2-factor MANOVA revealed a significant interaction between the effects of elevated CO_2_ on the performance characteristics of predators and prey (Pillai’s trace _(3,52)_ = 7.50, p<0.0001). ANOVA detected a significant interaction in the CO_2_ levels of predators and prey for six of the tested behavioral attributes: attack rate, predation rate, predator attack distance, reaction distance, apparent looming threshold and prey escape distance (see Table 2).

**Table 2:** Comparison of the effects of elevated CO_2_ (440, 880 µatm) on interactions between a predator (*Pseudochromis fuscus*) and prey (*Pomacentrus amboinensis*) on 6 performance variables: (a) attack rate (b) predation rate (c) predator attack distance (d) prey reaction distance (e) ALT and (f) prey escape distance.

behavior	source of variation	*df*	MS	*F*		*P*
**(a) attack rate**	predator	1	0.0042	0.143		0.705
	prey	1	0.0070	0.238		0.626
	predator*prey	1	0.1427	4.845		**0.030**
	error	70	0.0294			
**(b) predation rate**	predator	1	0.0049	0.464		0.829
	prey	1	0.1459	1.358		0.247
	predator*prey	1	0.4387	4.083		**0.020**
	error	67	0.1074			
**(c) predator attack speed**	predator	1	2017	0.213		0.646
	prey	1	38512	4.070		**0.048**
	predator*prey	1	61256	6.474		**0.013**
	error	56	9462			
**(d) prey reaction distance**	predator	1	685.5	0.422		0.519
	prey	1	8898.2	5.471		**0.022**
	predator*prey	1	16555.2	10.179		**0.002**
	error	56	1626.4			
**(e) ALT**	predator	1	228	0.011	0.916	
	prey	1	12548	0.616	0.435	
	predator*prey	1	313294	15.396	**<0.001**	
	error	54	20349			
**(f) prey escape distance**	predator	1	57241	6.365		**0.014**
	prey	1	40388	4.491		**0.038**
	predator*prey	1	69008	7.674		**0.007**
	error	56	0.4445			

Elevated CO_2_ significantly affected predator attack rates, however, this was influenced by prey CO_2_ exposure (Table 2). There was no difference in attack rate when predators and prey were exposed to the same CO_2_ treatments. However, when predators were exposed to elevated CO_2_, they displayed a significantly decreased attack rate against prey that had not been exposed to elevated CO_2_. Similarly, predators exposed to present-day CO_2_ levels had a reduced attack rate when paired with prey that had been exposed to elevated CO_2_ levels.

Exposure to elevated CO_2_ significantly affected predation rates (Table 2; Fig. 1b). For predators, exposure to elevated CO_2_ resulted in a significant reduction in the predation rates of prey that had not been exposed to elevated CO_2_. Post-hoc tests yielded a border line p-value (p = 0.05) for the comparison between the elevated CO_2_ exposed predators that interacted with present-day CO_2_ exposed prey and the elevated CO_2_ exposed predators that interacted with similarly exposed prey. Furthermore, when predators exposed to elevated CO_2_ were paired with similarly exposed prey, there was a significant increase in predation rates.

**Figure 2:**
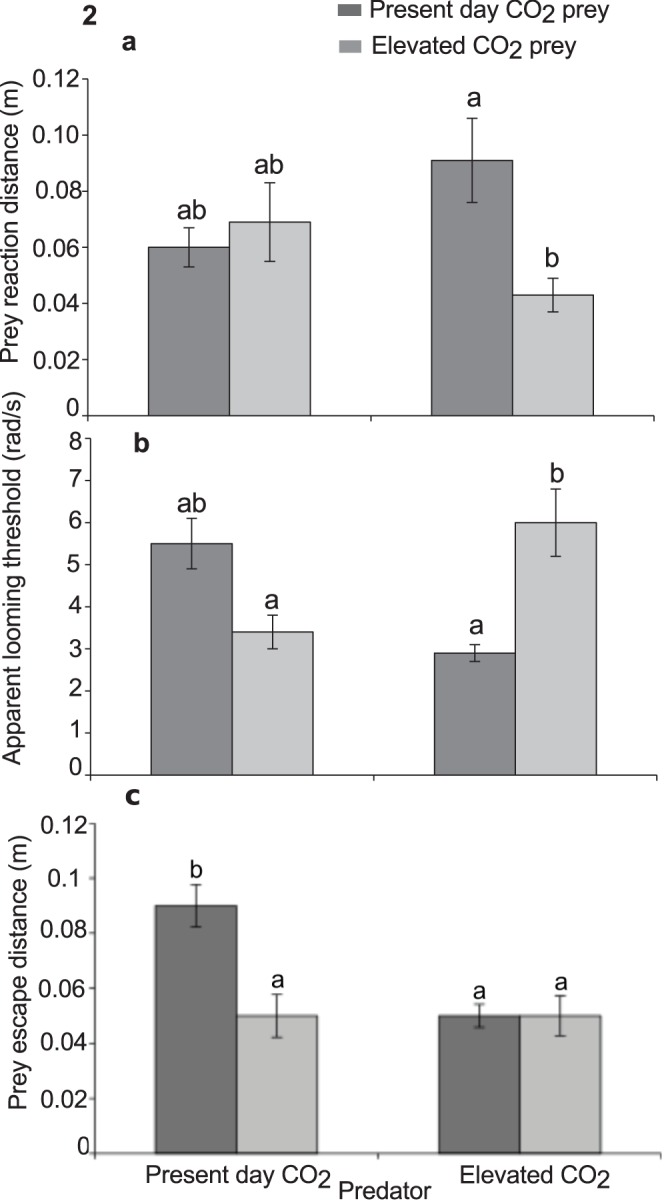
Effects of elevated CO_2_ on prey escape performance. Comparison of the effects of elevated CO_2_ (440, 880 µatm) on interactions between a predator (*Pseudochromis fuscus*) and prey (*Pomacentrus amboinensis*) on 3 performance variables of the prey: (a) prey reaction distance (b) apparent looming threshold (ALT) and (c) prey escape distance. (a) n = 19, 14, 11, 19 (b) N = 17, 14, 11, 16 and (c) N = 17, 14, 11, 18 (left to right). Errors are standard errors. Letters above bars represent Tukey’s HSD groupings of means.

Elevated CO_2_ significantly affected predator attack distance, but the nature of the effect differed according to whether prey had been exposed to elevated CO_2_ or not (Table 2; Fig. 1c). There was no effect of CO_2_ on attack distance when prey encountered control predators, however, when predators had been exposed to elevated CO_2_ they displayed significantly increased attack distances against prey that had also been exposed to elevated CO_2_ compared to control prey. The reaction distance of the prey to the predator at the onset of the first attack was affected by the CO_2_ treatment of the prey and predator (Table 2; Fig. 2a). Prey exposed to elevated CO_2_ allowed CO_2_ exposed predators to get closer to them before undertaking an escape response. Max predator speed was found not to differ between treatments.

Apparent looming threshold (ALT) was significantly affected following exposure to elevated CO_2_. When prey that had been exposed to elevated CO_2_ were paired with similarly exposed predators, ALT increased significantly. In contrast, when control prey were paired with CO_2_ exposed predators, ALT decreased substantially. There was no significant difference in ALT between the crossed trials (Table 2; Fig. 2b).

Prey escape distance was also found to be significantly affected following exposure to elevated CO_2_ (Table 2; Fig. 2c) with prey exposed to present-day CO_2_ having the highest escape distance compared to the other three treatment combinations.

Prey escape speed during stage 1 and 2 and maximum prey escape speed were not influenced by the exposure of either prey or predator to elevated CO_2_ and no interactions between predator and prey treatments were found.

## Discussion

Although recent studies have demonstrated that exposure to elevated CO_2_ significantly increases prey mortality rates [9], [28], [38], the effects of elevated CO_2_ on the kinematics at the basis of predator-prey interactions has not been investigated. Here, we demonstrated that CO_2_ levels that may occur in the surface oceans by the end of the century impact on both the kinematics and the timing of predator-prey interactions. More specifically, prey treated with elevated CO_2_ showed changes in escape performance, such as shorter reaction distances, reduced escape distances and changes in apparent looming threshold (ALT). Because CO_2_ exposed prey were closer to the predator at the onset of their escape reaction and they swam shorter distances, elevated CO_2_ had a clear negative effect on the reactivity and locomotion performance of the prey. The fast kinematics of the escape responses are likely to be under the control of Mauthner cells (although control by other reticulospinal cells cannot be ruled out [39] ), which are triggered as a reaction to the fast approach of a predator. It is therefore possible that increased CO_2_ levels may have an intrinsic effect on the sensory performance and neural control by the Mauthner cells or other reticulospinal neurons at the basis of the escape response, leading to increased prey vulnerability and ultimately increased mortality. Because escape distance is affected by CO_2,_ it is possible that CO_2_ affects mainly the motivational component of the motor response, which acts upon the duration of the burst. Nevertheless, the effects of CO_2_ on the onset of the response (i.e. reaction distance), suggest that the sensori-motor performance and the timing of the Mauthner cell’s firing are also affected. Previous work has demonstrated that the regulation of plasma and cellular HCO_3_^−^ and Cl^−^ following exposure to high CO_2_ may lead to the excitation of GABA-A receptors [18]. Given that GABA-A receptors are found throughout the Mauthner neuron [40], these results suggest that high CO_2_ interferes with GABA-A receptor function resulting in the misfiring of action potentials. Therefore, it is possible that this interference with brain neurotransmitters may be responsible for the changes observed. These results are consistent with the increased mortality rates observed in CO_2_-exposed prey [9], [28] and suggest that higher vulnerability to predation may be caused by a combination of changes in escape performance and other behavioural traits, such as increased activity levels and distance from shelter.

The results for the predators are complex to interpret, because the overall predation rate is the result of both predator performance during the attack and its motivation to attack. Attack rates and attack distances were affected by elevated CO_2_, with the lowest attack rates and distances occurring when predators and prey had experienced different CO_2_ histories. This demonstrates that high CO_2_ has an effect on predator performance during a predator-prey encounter, however the extent of this effect appears to be dependent upon the extent to which prey are affected by high CO_2_. Predatory attacks require not only high speed but also precision to aim at the prey. The neural mechanisms that control predatory attacks are poorly understood, although it has been suggested that in some cases they may also be controlled by Mauthner cells [41]. It is possible that future levels of high CO_2_ will impact on the complex circuitry needed to carry out attack processes. This may explain why we see behavioural changes that result in either decreased or increased capture success when predators and prey are exposed to different CO_2_ histories, but when both are treated similarly, these changes appear to ‘level out’ resulting in no change in overall capture success compared with controls. Similar results have been suggested in estuarine systems involving crustaceans and molluscs, where for individual species there was a negative effect following CO_2_ exposure, but these affects were not manifest at the community level (i.e. within the predator-prey interaction) [42]. This suggests that elevated CO_2_ will have a marked impact on the dynamics and outcome of predator-prey encounters, but the population and community-level effects are likely to be dependent on the sensitivity of species to elevated CO_2_.

This is the first study to examine performance-based attributes of both predators and prey under a high CO_2_ environment within the context of a predator-prey interaction. While our findings are specific to the species used, the fact that the behavior of both predator and prey were affected is strongly suggestive that elevated CO_2_ will affect the behavior of other predator and prey fish. Whether in other cases the increase in CO_2_ will provide an advantage for the prey, the predator or neither, will depend on the extent to which the specific predators and prey individuals are tolerant to CO_2_ changes. Recent studies have shown that effects of elevated CO_2_ can differ markedly between species [9]. Moreover, the effects of elevated CO_2_ are not necessarily restricted to single fish-fish interactions, but might be manifest through other trophic pathways.

Changes in seawater *p*CO_2_ has been shown to impact fishes at all stages of development [10], [43], with significant impacts on individual fitness occurring at the vulnerable juvenile stage. Alterations to the dynamics of regulating processes at this early life stage could have significant affects on the replenishment and sustainability of marine populations [28]. In addition to affecting the physiology and behavior of given species, elevated CO_2_ is likely to influence interactions between species, including predator-prey encounters. This may have important ecological effects, such as changing the balance of an interaction in favor of the predator or the prey. This study highlights the importance of considering species interactions when making predictions concerning the response of communities to climate change [15], [44].
